# Machine Learning Classification Model Performance in Detecting Cognitive Impairments From Multimodal Embeddings

**DOI:** 10.1093/geroni/igaf122.1204

**Published:** 2025-12-31

**Authors:** W Quin Yow, Adharsha Sam Edwin Sam Devahi, Lihan Zuo, Ka Lon Sou

**Affiliations:** Singapore University of Technology and Design, Singapore, Singapore; Singapore University of Technology and Design, Singapore, Singapore; Singapore University of Technology and Design, Singapore, Singapore; Singapore University of Technology and Design, Singapore, Singapore

## Abstract

In the fast-evolving field of AI for healthcare, there is a growing trend of combining acoustic and linguistic features from speech data to improve ML model performance for cognitive impairment detection. This paper evaluates a hyperparameter-tuned SVM model trained on three feature sets derived from pretrained Transformer-based models: acoustic CrisperWhisper embeddings, linguistic BERT embeddings, and both. Using 150 data points (94F, ages 51-99) equally distributed across healthy, MCI, and dementia classes, sourced from DementiaBank and challenge datasets from the University of Edinburgh and Carnegie Mellon University, no statistically significant differences (χ²=3.45, p = 0.178) were found in the accuracy scores of the model trained on the three feature sets across 30 validation datasets. The mean validation accuracy scores of the model trained on acoustic, linguistic, and combined embeddings were 72% (SD = 0.233), 63% (SD = 0.221), and 57% (SD = 0.202), respectively. However, there were statistically significant differences (χ²=58.067, p < 0.001) in the accuracy scores of the model trained on the feature sets across 30 training datasets. Among significant pairwise comparisons, the training accuracy scores of the model trained on acoustic embeddings (M = 1, SD = 0) were significantly higher than those using linguistic embeddings (M = 0.847, SD = 0.008; W = 0, n = 30, p < 0.001) and those using acoustic+linguistic embeddings (M = 0.875, SD = 0.012; W = 0, n = 30, p < 0.001). The results suggest that while acoustic embeddings alone yielded the highest accuracy, hyperparameter tuning failed to mitigate overfitting, and acoustic+linguistic embeddings did not improve model performance. However, given the small dataset size, these findings should be interpreted with caution. Future directions for improving model performance will be discussed during the presentation.

